# How does age affect baseline screening mammography performance measures? A decision model

**DOI:** 10.1186/1472-6947-8-40

**Published:** 2008-09-21

**Authors:** John D Keen, James E Keen

**Affiliations:** 1 Department of Radiology, John H. Stroger Jr. Hospital of Cook County, 1901 West Harrison Street, Chicago, IL. 60612-9985, USA; 2 Department of Veterinary and Biomedical Sciences, University of Nebraska, P.O. Box 148, Clay Center NE 68933, USA

## Abstract

**Background:**

In order to promote consumer-oriented informed medical decision-making regarding screening mammography, we created a decision model to predict the age dependence of the cancer detection rate, the recall rate and the secondary performance measures (positive predictive values, total intervention rate, and positive biopsy fraction) for a baseline mammogram.

**Methods:**

We constructed a decision tree to model the possible outcomes of a baseline screening mammogram in women ages 35 to 65. We compared the single baseline screening mammogram decision with the no screening alternative. We used the Surveillance Epidemiology and End Results national cancer database as the primary input to estimate cancer prevalence. For other probabilities, the model used population-based estimates for screening mammography accuracy and diagnostic mammography outcomes specific to baseline exams. We varied radiologist performance for screening accuracy.

**Results:**

The cancer detection rate increases from 1.9/1000 at age 40 to 7.2/1000 at age 50 to 15.1/1000 at age 60. The recall rate remains relatively stable at 142–157/1000, which varies from 73–236/1000 at age 50 depending on radiologist performance. The positive predictive value of a screening mammogram increases from 1.3% at age 40 to 9.8% at age 60, while the positive predictive value of a diagnostic mammogram varies from 2.9% at age 40 to 19.2% at age 60. The model predicts the total intervention rate = 0.013*AGE^2 ^- 0.67*AGE + 40, or 34/1000 at age 40 to 47/1000 at age 60. Therefore, the positive biopsy (intervention) fraction varies from 6% at age 40 to 32% at age 60.

**Conclusion:**

Breast cancer prevalence, the cancer detection rate, and all secondary screening mammography performance measures increase substantially with age.

## Background

Analysts have debated the benefits and harms of screening mammography in the medical literature for over a decade [1-3]. The 1997 National Institutes of Health Consensus Conference concluded that younger women should decide for themselves whether and when to begin screening. Physicians must therefore understand breast cancer risk and the accuracy and consequences of screening in order to help women make that decision [4]. Analysts, advocates and critics of screening mammography acknowledge the need for informed medical decision-making (IMDM) [5-7]. Recently, the American College of Physicians has advocated informed decision-making concerning screening mammography for women under 50 [8]. However, analysts have not applied the tool of decision analysis to study the initial or baseline screening mammogram.

Women desire accurate information from their physicians about the benefits, limitations, and potential harms of screening mammography before their baseline exam. Furthermore, 90% want involvement in the screening decision [9], while 80% are confident in their decision-making ability if presented with relevant information [10]. The fact that this information has not been effectively communicated raises ethical questions regarding the biased promotion of screening mammography [11]. For example, in one survey 60% of U.S. women believed that mammography prevents or reduces the risk of contracting breast cancer, rather than the risk of dying from breast cancer [12]. In reality, the development risk for breast cancer is probably higher with mammography due to ionizing radiation [5,13]. Two studies showed that only around 5% of women are aware of the existence of ductal carcinoma in situ (DCIS) or nonprogressive cancers [14,15]. Furthermore, 85% of women think that mammography seldom misses cancers [15].

Since a woman's decision to obtain a baseline mammogram is an independent first step in the screening process, we used decision analysis to model the possible outcomes of the baseline screening mammogram as a starting point to help educate women and promote consumer-oriented medical care. A recent Institute of Medicine report stressed the importance of publicizing three screening mammography performance measures, including the cancer detection rate (CDR), the recall rate, and the positive biopsy fraction (PBF) [16]. Consequently, we used the decision model to predict how these primary events and secondary performance measures change as a function of age.

## Methods

### Decision model

Screening mammography by definition involves only women without any breast symptoms that might indicate that cancer is present [17]. A baseline mammogram is the first screening mammogram a woman obtains. The radiologist reading the mammogram looks for evidence of breast cancer including masses and small calcifications. If no suspicious findings are present, the radiologist recommends routine screening to check for the interval development of cancer. The next screening mammogram a woman obtains is a subsequent mammogram, which the radiologist compares with the baseline mammogram if available. If the radiologist sees suspicious findings on a baseline or subsequent screening mammogram, the woman returns for a diagnostic mammogram or ultrasound to determine if the findings are more likely from breast cancer or more likely from normal variation. After the diagnostic imaging, the radiologist may recommend that the woman return to routine screening, receive a short-term follow-up mammogram, or consult with a breast surgeon for clinical evaluation and possible image-guided or surgical biopsy of the suspicious findings on the mammogram.

**Figure 1:**
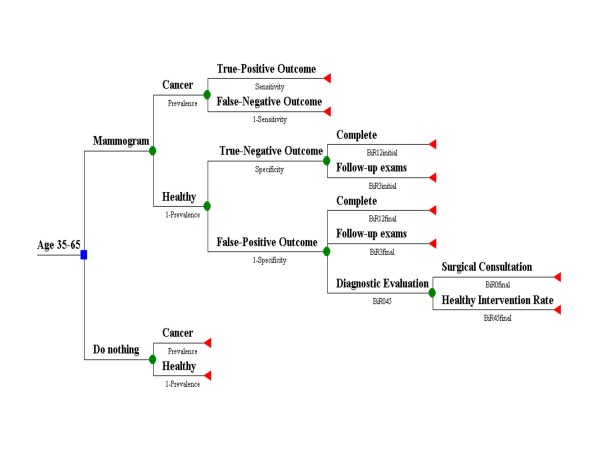
**Decision model for baseline screening mammography used to predict primary and secondary performance measures**. An asymptomatic woman between ages 35 and 65 getting a baseline mammogram faces four possible outcomes. These outcomes depend on her health status (cancer or healthy) and the initial radiologist reading (positive or negative). The corresponding inputs for the circular probability nodes are population-based values of age-dependent cancer prevalence and radiologist accuracy. There is a wide range of radiologist performance for sensitivity and specificity. The probability of a true-positive outcome is equivalent to the cancer detection rate, a screening benefit. The false-positive outcome involves healthy women and is a screening harm. Besides additional diagnostic imaging, this outcome may result in further diagnostic evaluation and intervention. The probability values for the third and fourth branch points (BiR12initial, etc.) use population-based data.

Decision analysis applies quantitative methods to help optimize decision making under uncertainty, with applications in diagnostic radiology [18]. We created a decision tree that models a simplified screening scenario starting with the choice that a woman faces starting in her late thirties: either to start screening for breast cancer and begin with a baseline screening mammogram, or postpone screening by doing nothing, as shown by the decision node square in Figure 1. The first branch point (probability node circles) of each decision splits into women with and without cancer, while the second branch point for those getting screening splits into a positive or negative mammogram, with terminal outcome nodes (triangles) for those with cancer. The third branch point includes diagnostic and subsequent imaging, consultations, and interventions for healthy women. We did not include likely stages of diagnosis or the effects of overdiagnosis since they do not affect the calculation of the performance measures. To keep the model simple and easier to comprehend, we did not simulate the effects of repeat screening. We used DATA™ 3.5 software (TreeAge, Williamstown, MA, 1999) and Excel (Microsoft, Redmond WA, 2003) to construct the computer model.

To summarize the model inputs, the first branch point probability node uses age-dependent population-based estimates for breast cancer prevalence. We derived the prevalence values from the Surveillance Epidemiology and End Results (SEER) program age-specific incidence rates 1998–2002 [19], as shown in Table 1[20-23]. The second branch point probability nodes use population-based estimates for screening mammography diagnostic accuracy (sensitivity and specificity) as the major input. Table 2 shows the most recent relevant published accuracy data from the Breast Cancer Surveillance Consortium (BCSC) [24-26], which are derived by using the Breast Imaging Reporting and Data System (BI-RADS) categories [27]. We used published intermediate and final outcomes specific to baseline exams for the healthy women third branch point probability nodes, while we derived the fourth branch point probability node value from published total biopsy rates. We describe all derivations in detail at the end of the Methods section.

**Table 1:** Breast cancer incidence rates and estimated prevalence.

**Age**	Invasive*	**DCIS^†^**	**Total**	**DCIS**	**Sojourn**	**Prevalence^‡^**	**Low^§^**	**High**
	Annual incidence per 1000 women	Annual incidence per 1000 women	Annual incidence per 1000 women	% of all breast cancer	Years	Cases/1000		

**35.0**	0.44	0.07	0.51	14	2.0	1.03		
**37.5**	0.62	0.11	0.73	15	2.0	1.46		
**40.0**	0.91	0.24	1.15	21	2.0	2.30		
**42.5**	1.20	0.36	1.57	23	2.1	3.24	2.9	3.7
**45.0**	1.58	0.48	2.06	23	2.2	4.39	3.9	4.9
**47.5**	1.95	0.60	2.55	24	2.4	6.08	5.3	7.0
**50.0**	2.25	0.70	2.95	24	2.7	7.78	6.7	9.0
**52.5**	2.56	0.80	3.36	24	2.9	9.68	8.1	11.3
**55.0**	2.96	0.86	3.82	23	3.1	11.97	9.9	14.1
**57.5**	3.36	0.92	4.28	22	3.4	14.56	12.1	17.2
**60.0**	3.65	0.95	4.60	21	3.7	16.86		
**62.5**	3.95	0.97	4.92	20	3.9	19.34		
**65.0**	4.18	1.00	5.18	19	4.2	21.76		

* Incidence rate data from SEER 1998–2002. [19]

^† ^Ductal carcinoma in situ.

^‡ ^Prevalence is incidence rate (Invasive + DCIS) times sojourn time. [20-23]

^§ ^Low and high ranges apply the low and high values for sojourn time.

**Table 2:** Breast Cancer Surveillance Consortium (BCSC) recent studies of screening mammography accuracy.

**Parameter**	**Value^†^**	**Range^‡^**	**Ages**	**Period^§^**	**Previous exams**	**Years**
Sensitivity*	71.3%		< 40	1	No	1996–01
	53%			2		
	82.1%		40–49	1	No	
	61%			2		
	92.1%		50–59	1	No	
	68%			2		
	89.3%		60–69	1	No	
	66%			2		
	68.2%		< 40	1	Yes	
	70.7%		40–49	1	Yes	
	78.1%		50–59	1	Yes	
	79.7%		60–69	1	Yes	
	88.6%	85.8–91.4	40–89	1	No	1996–00
	76.8%	75.7–77.9	40–89	1	Yes	
	77.8%	77.6–78.0	40–89	1	Both	
	65.3%		40–89	2	None past 5 yrs	1996–99
	53.8%		40–89	2	Yes	
Specificity^||^	85.9%	85.6–86.1	40–89	1	No	1996–00
	92.5%	0.0	40–89	1	Yes	
	92.1%	91.9–92.3	40–89	1	Both	
	90.5%		40–89	2	None past 5 yrs	1996–99
	93.3%		40–89	2	Yes	

* Positive exam is Breast Imaging Reporting and Data System (BI-RADS)[27] category 0, 4, 5, and category 3 if there is immediate diagnostic evaluation.[26]

^† ^The first four age-dependent one-year baseline sensitivity values are from reference [25]. The matching two-year values also used in the model are derived (see text). Matching one-year previous mammography sensitivity values are provided for comparison from reference [25].

^‡ ^Ranges are 95% confidence intervals available only for one-year accuracy values ages 40–89 from reference [26].

^§ ^Time frame for calculating sensitivity and specificity. Two-year accuracy values for ages 40–89 are from reference [24].

^|| ^The one-year specificity values are from reference [26]. The baseline specificity value is used in the model for all age groups.

The screening mammogram can have four possible outcomes: true-positive, false-negative, true-negative, and false-positive, from which the model predicts the two primary events CDR and recall rate. For example, the recall rate includes all women called back for additional imaging evaluation based on the initial radiologist reading of a positive screening mammogram, and these exams could be true-positive (cancer) or false-positive (healthy). The model calculates the predicted secondary performance measures using the primary events and the distal branches of the false-positive outcome screening mammogram.

### Model predictions: performance measures

As shown in Figure 1, screening mammograms in women with cancer (D+) are classified as either true-positive cases (TP) or false-negative cases (FN), so D+ = TP + FN. Mammograms in healthy women (D-) may be either true-negative cases (TN) or false-positive cases (FP), so D- = TN + FP [28,29]. The probability of a true-positive outcome for all screened women, shown as the top 3^rd ^division branch of the decision tree in Figure 1, equals sensitivity (TP/(TP+FN)) times prevalence (a proportion, or D+/1000 screens), or TP cases/1000 screens. We can also define the sensitivity as the true-positive fraction, or TPF [30]. Likewise, the probability of a false-positive outcome or FP cases/1000 screens equals [1 – specificity] times [1 – prevalence]. Overall specificity is defined by the true-negative fraction, or TN/(TN + FP), with the complement the false-positive fraction (FPF), or 1-specificity = FP/(TN +FP). In our model, the sum of the probabilities of true-positive outcomes, false-negative outcomes, true-negative outcomes, and false-positive outcomes is equal to one.

We calculated the primary events including the CDR and the recall rate from the four possible mammography outcomes as follows. The CDR, defined as cancers detected per 1000 women screened [16], is equivalent to the probability of a true-positive outcome, or TP cases/1000 screens. The BCSC definition of CDR = TP/(TP + FN + TN + FP), where the denominator equals all cases or all women screened (D+ plus D-) [26]. In our model, the recall rate or abnormal interpretation rate equals the sum of the initial radiologist readings of positive cases/1000 screens [16], or the probability of a true-positive outcome plus the probability of a false-positive outcome. We can convert the recall rate to a percentage of all screens by dividing the numerator by ten.

We calculated the secondary performance measures including predictive values as follows. The positive predictive value of a screening mammogram (PPVS) is a proportion (TP/(TP + FP)) [16], and equals the CDR divided by the recall rate [31]. Likewise, the negative predictive value of a screening mammogram is a proportion that equals the TN cases/1000 screens (specificity times [1-prevalence]), divided by the sum of the negative cases/1000 screens. The positive predictive value of a diagnostic mammogram (PPVD) following an abnormal screening mammogram is the CDR divided by the sum of the CDR and the healthy women diagnostic evaluation rate (a percentage of the probability of a false-positive outcome, the bottom 3^rd ^division branch). The total intervention rate (TIR) is the healthy women intervention rate (a percentage of the diagnostic evaluation rate) plus the CDR (women with cancer intervention rate). Finally, the PBF is the CDR divided by the total intervention rate.

To simplify our results in order to support IMDM, we generated scatter plots of incidence and prevalence and each model prediction versus age in Excel (Microsoft, Redmond WA, 2003). We determined the best-fitting trend line by simple linear regression as defined by highest coefficient of variation (R^2^) for each model outcome, with a linear fit for recall rate and negative predictive value for simplicity. For comparison purposes, we took available updated but unpublished data from the BCSC website (available at ) for women with no previous mammography from 1996 to 2005 for 5-year age groups 40–44 through 60–64 including the CDR, recall rate, and PPVS. We used the BCSC cancer rate by age to compare with our input prevalence [32]. We also used the most recently published BCSC data for the CDR, recall rate and PPVS for age groups 40–49 and 50–59 [25].

### Sensitivity analysis

To estimate the effect of radiologist variability including the callback threshold (sensitivity/specificity pair) as well as skill level, we calculated a range of values for the model output. We took the BCSC radiologist performance sensitivity and specificity at the 10^th ^and 90^th ^percentiles and adjusted our values by the ratio of these outliers to the median [32]. For example, the BCSC 90^th ^percentile for sensitivity is 89.3; the median is 81.3, giving a ratio of 1.0984. Likewise, the 10^th ^percentile ratio is 0.8303 (67.5/81.3). For specificity, we adjusted the FPF, or 1-specificity, in a similar manner, so that the 90^th ^percentile ratio of performance is 0.481 (5.1/10.6), while the 10^th ^percentile ratio is 1.632 (17.3/10.6). We assumed these ratios were constant across all ages. Therefore, the CDR directly reflects variation in sensitivity. However, since the recall rate and all other performance measures use both the CDR and the FPF, we assumed that a reader with low specificity (high FPF) would also read at high sensitivity (high TPF), as supported by BCSC data [33].

### Model inputs: probability nodes

#### First branch point: prevalence

Prevalence is the percentage of the population possessing a disease at a given point in time. The incidence rate is the number of new cases that develop over a specific period. A baseline exam will identify undetected prevalent disease. Prevalence approximately equals the incidence rate times the average duration of disease [20]. Assuming screening could start somewhere between ages 35 and 65, we derived prevalence input data for the model using the SEER program age-specific incidence rates for both DCIS and invasive cancer from 1998 to 2002 [19]. In the case of screening of asymptomatic women, the average duration of disease is the preclinical mammography detectable state, or the period of time (sojourn) during which imaging can theoretically detect a cancer but before a woman has symptoms [21].

We assumed an average sojourn time by combining estimates of sojourn time from three screening mammography trials including Canada, Stockholm, and Swedish Two-County. We calculated an estimate of 2.13 years for ages 40–49 ((1.9+2.1+2.4)/3), and 3.13 years for ages 50–59 (2.6+3.1+3.7)/3), and the single value of 4.2 years for ages 60–69 [22,23]. We applied these values at ages 45 and 55 and 65 and used linear interpolation to derive the remaining values. We also assumed a base value of 2.0 for ages 35–40. Under these assumptions, Table 1 shows the calculated breast cancer prevalence per 1000 women. For comparison purposes, we calculated a reasonable low and high range for prevalence using the range of sojourn times at 45 and 55 and repeated interpolation. However, we did not vary the model input prevalence due to lack of ranges for younger and older women and in order to emphasize the effect of radiologist accuracy on the model predictions.

#### Second branch point: accuracy

Table 2 includes baseline and subsequent screening mammography sensitivity and specificity from the BCSC published literature, which reflects the United States population. We considered a screening mammogram a positive test result (versus negative) if the radiologist's assessment led to immediate further evaluation, which includes BI-RADS category 0, 4, or 5, or a 3 with recommendation for additional diagnostic imaging or surgical consultation [26]. For BI-RADS audit comparison purposes, mammography sensitivity is typically (and arbitrarily) defined as the number of patients diagnosed with cancer within one year of a positive mammogram, divided by all screened patients with a diagnosis of cancer over one year [27]. From a practical standpoint, less than 5% of United States mammography facilities have the means to keep track of false-negative results by linking mammograms to breast cancers [16]. However, the BCSC has this ability and uses the BI-RADS audit definitions of accuracy when collecting its data [31].

We also derived a two-year sensitivity because two years is both close to the sojourn time period of preclinical detectability and the time interval recommended for screening by an advisory panel [34]. Two years gives more time for cancers not identified at mammography (assuming they were detectable) to present themselves, so the two-year sensitivity will be lower [35]. However, some slower-growing cancers present at the initial screening mammogram will not become apparent. Therefore, we can make an argument for a three-year sensitivity based on sojourn times for women over 50, but as the measuring period increases, the chance increases that faster-growing cancers that were not detectable would be counted as not identified.

We derived the age-dependent two-year sensitivity by using the most recently published BCSC age-dependent one-year values of 71–92% for women with no previous mammogram shown in Table 2[25]. We multiplied this value by the two-year to one-year sensitivity ratio using BCSC data for women ages 40–89, or 65.3/88.6 = 74%. This ratio is close to the Canada, Stockholm, and Swedish Two-County screening mammography trials first-round screen ratio of ((0.67+0.79+0.91)/3), or 79% [36]. Application of the BCSC ratio gives a two-year sensitivity value of 53% (0.74*71.3) for women under age 40, 61% (0.74*82.1) for women ages 40–49, 68% (0.74*92.1) for women ages 50–59, and 66% (0.74*89.3) for women ages 60–69. Comparison with previous BCSC studies shows that sensitivity has improved over time and increases with age for first screening and subsequent mammography [37,38]. For subsequent mammography, sensitivity also increases with lower breast density [39]. Table 2 shows sensitivity for BCSC subsequent mammography is about 85% (54/65, 77/89) of first screening sensitivity, likely due to harder-to-detect smaller tumors [36].

For specificity, we used the BCSC one-year value of 85.9% for women ages 40–89 (95% CI 85.6–86.1), which is based on 70,200 first (no previous mammography) mammograms, with 64% of the women under age 50 [26]. Specificity with first screening mammography is stable with age, and for subsequent mammography increases slightly from 91% at 40–49 to 93% at 60–69 [38,39]. Furthermore, the one-year specificity value should not vary much with increasing time with low prevalence of disease [35]. The BCSC recall rate of 14.7% (95% CI 14.4–14.9) includes the true-positive and false-positive exams, or (TP + FP)/(TP + FN + TN + FP) [26]. With low prevalence typical in screening mammography (under 2%), the FPF or (FP/(TN +FP)) is very close to the recall rate since TP and FN are relatively small.

#### Third branch point: intermediate outcomes

Figure 1 shows that women with cancer have the cancer detected (true-positive) or not (false-negative) depending on the sensitivity. Healthy women have true-negative or false-positive mammograms with possible additional evaluation. We derived probabilities for these outcomes based on BCSC data for 119,000 first mammography exams (no history of or over 5 years since last mammogram) [24]. Within the true-negative outcome (85.9% (specificity) of healthy women), 96% would be complete (BiR12initial probability), while 4% would face follow-up exams for stability of the findings in 6 months and 1 and maybe 2 years (BiR3initial probability). For the false-positive outcome (14.1% of healthy women), women would be recalled by the radiologist within 90 days to undergo additional diagnostic imaging, usually unilateral additional view mammography and/or ultrasound. We consider this evaluation after a positive screening examination an "indirect" diagnostic mammogram, while a "direct" diagnostic mammogram follows from the evaluation of a symptomatic breast problem and would have its own PPVD [40]. After this diagnostic imaging evaluation, 38.2% would be categorized as complete (BiR12final probability), 16.2% would face follow-up exams (BiR3final probability), and 45.6% would undergo additional diagnostic evaluation or consultation (BiR045 probability), or 6.4% (14.1*45.6) of the women in the false-positive branch.

#### Fourth branch point: intervention rate

We assumed 50% of these healthy women would have a surgical consultation followed by a clinical follow-up examination (BiR0final probability). The other 50% (BiR45final probability) would have a surgical consultation followed by an intervention, including tissue biopsy or fine-needle aspiration (FNA). This probability assumption allows correlation with published total (cancer and benign) intervention rates and predicts a healthy women (benign) intervention rate after baseline screening of 32.1/1000 at age 40 to 31.6/1000 at age 60. We decided to use the PBF instead of the positive predictive value of a recommended biopsy (BI-RADS 4 and 5), since we assumed women would care more about actual chances of an intervention. In actual practice, about one-third of BCSC tracked biopsies occur after a BI-RADS 1, 2 or 3 classification [41]. We defined the PBF denominator to include all interventions, including FNA.

We obtained total intervention rates from the BCSC and the National Breast and Cervical Cancer Early Detection Program (NBCCEDP) for first screening mammograms, the latter targeting low-income women. In one analysis of both programs from 1996 to 1999, first screening mammograms were described as mammograms among women with no previous mammogram in the database and no self-reported mammogram within 5 years. Recommended biopsy rates were 24.4/1000 (95% CI 23.0–26.0) for BCSC first screening mammograms for women over age 50 and were 9.9/1000 (95% CI 9.7–10.2) for subsequent mammograms, for a first/subsequent ratio of 2.4. The recommended rates and the total open biopsy fraction increased slightly with age. The results for the NBCCEDP were 31/1000 (95% CI 30–32) [42]. These results included FNA, so the recommended biopsy rate equals the recommended intervention rate.

The BCSC total recommended tissue biopsy rate from 1996 to 2001 was 12.7/1000, following mostly subsequent (93%) but including baseline mammograms. The biopsies reported and performed within one year were 16.1/1000, giving a reported/recommended tissue biopsy ratio of 1.27 (16.1/12.7). These tissue biopsy rates were constant for ages 40–89. Since about 32% of the BCSC recommended biopsies had no pathology results reported, although they were likely performed (or had FNA), true intervention rates are likely higher than the reported performed results of 16.1/1000 by an additional (12.7/1000 *0.32) = 4.1/1000 [41]. This means true intervention rates could be from 16.1/1000 (reported) to 20.2/1000 (reported plus assumed performed), giving a ratio of 1.25. The estimated PBF is 15.6% (1612/10346) at ages 40–49 and 26.6% (2470/9283) at 50–59 if we apply the 1.25 multiplier to the benign tissue biopsies. Finally, we multiplied the subsequent mammography intervention rates by the first/subsequent ratio of 2.4 for women over 50 to estimate the total intervention rate for a baseline mammogram of 38–48/1000.

We also used an updated analysis of the NBCCEDP from 1995 to 2002, which excluded women with symptoms (about 11%) in a subset of the analysis. There were 703,000 baseline exams, defined as first program screening mammograms regardless of previous reported history of mammography. For example, the CDR for ages 40–49 was 4.0/1000 and the total biopsy rate (without FNA) was 26.1/1000 [43], which would increase to 31.3/1000 assuming a multiplier of 1.2 for FNA was applied. This multiplier is from an academic medical center with an overall intervention rate of 16.8/1000 [44]. Therefore, taking the CDR and dividing by the intervention rate gives a PBF of 12.8% (4.0/31.3) for 40–49, and values of 18.9% (5.3/28.1) for 50–59, and 26.7% (7.5/28.1) for 60–64. Including women with symptoms for ages 40–49 increased the CDR to 8.9/1000 and the total biopsy rate to 40.0/1000, which would limit the use of the NBCCEDP data for other performance parameters. The low-income status of the participants also would likely make these intervention rates less representative compared to an insured population.

## Results

**Figure 2:**
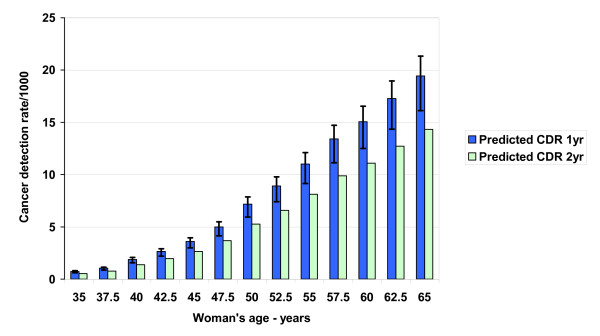
**Predicted baseline screening mammography primary performance measure: cancer detection rate (CDR)**. The probability of a true-positive outcome is equivalent to the CDR, or breast cancers detected per 1000 screening mammograms for women ages 35–65. We calculated the CDR using both one-year and two-year sensitivity estimates with no range assumed for prevalence. Sensitivity is the probability that a radiologist interprets a mammogram as positive in screened women who have a cancer diagnosis over one or two years of follow-up. The 90^th ^(high error bars) and 10^th ^(low error bars) percentile of radiologist performance for sensitivity generates the range around the one year predictions.

**Figure 3:**
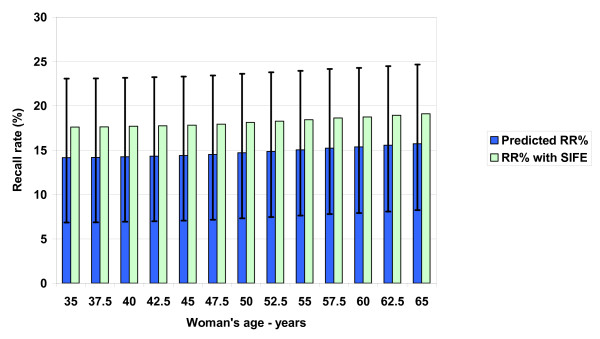
**Predicted baseline screening mammography primary performance measure: recall rate (RR)**. The RR percentage is recalls per 100 (not 1000) screening mammograms for women ages 35–65. Women both with and without cancer are recalled for further diagnostic imaging of abnormalities seen on the initial positive screening mammogram. The 10^th^/90^th ^(high error bars) and 90^th^/10^th ^(low error bars) percentile of radiologist performance for specificity/sensitivity generates the range around the RR predictions. Specificity is the fraction of negative mammograms in women who remain healthy over one year of follow-up. SIFE are short interval follow-up mammogram examinations without additional diagnostic imaging.

**Figure 4:**
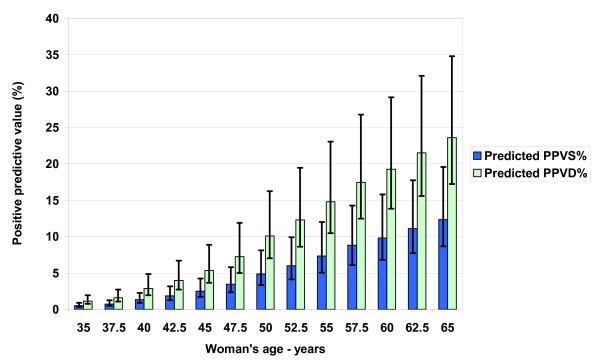
**Predicted secondary performance measures: positive predictive values for screening and diagnostic mammograms (PPVS, PPVD)**. Increasing cancer prevalence and screening sensitivity with age mean radiologists detect more cancers and the potential absolute benefit of baseline mammography increases with age. Recall exams are mostly false-positive mammograms, which cause unwanted collateral costs from screening. The relative stability of the recall rate makes the positive predictive value performance measures increase with age. The reciprocal of the positive predictive value is the number of screening recall mammograms or positive diagnostic mammograms needed to detect a cancer.

**Figure 5:**
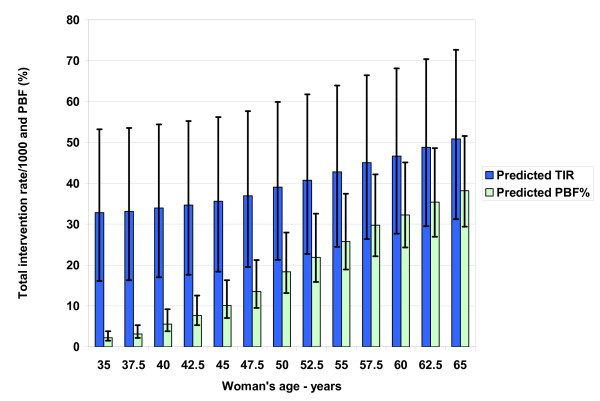
**Predicted secondary performance measures: total intervention rate and positive biopsy fraction (TIR, PBF)**. The TIR is for 1000 screening mammograms for women ages 35–65 and includes the interventions of tissue biopsy and needle aspiration. The PBF percentage is a widely accepted performance measure due to the desire to avoid the high financial and emotional cost and invasive nature of negative interventions. The model predicts that the PBF does not reach the minimum recommended level of 25% until age 55. Stratifying the recommendations for screening so that only higher risk women would be encouraged to screen at younger ages would increase the prevalence and therefore improve all performance measures.

Figures 2 and 3 show the model-predicted primary events (CDR and recall rate) associated with baseline mammography, while Figures 4 and 5 summarize predicted secondary performance measures. Table 3 shows the regression equations for incidence, prevalence and model predictions with the associated R^2 ^value. The increase in CDR with age generally tracks the prevalence 2^nd ^order polynomial, while the recall rate is linear and stable with age. Both the primary events and the secondary performance measures have a wide range of values depending on radiologist performance at the 10^th ^or 90^th ^percentiles. Specificity dominates the recall rate by an order of magnitude: the false-positive outcomes (140/1000) and recall rate (147/1000) at age 50 are about 20 times the CDR (7.2/1000). At age 50, the recall rate range from 10^th ^to 90^th ^percentile is 16.3%, but this decreases only to 16.1% when using a median sensitivity at the extremes of specificity. Therefore, equal absolute percentage changes in radiologist sensitivity or specificity would have disparate effects.

**Table 3:** Best-fit equations for model predictions.

Model Input*	**Equation**	**R^2^**
Incidence	y = 0.165*AGE - 5.37	0.996
Prevalence	y = 0.016*AGE^2 ^- 0.90*AGE + 12.54	0.998
**Model Output**		
Cancer detection rate 1 year	y = 0.013*AGE^2 ^- 0.70*AGE + 8.26	0.996
Recall rate	y = 0.054*AGE + 12.08	0.957
PPV^†^, screening	y = 0.007*AGE^2 ^- 0.31*AGE + 2.18	0.995
NPV^‡^, screening	y = -0.007*AGE + 100.2	0.814
PPV^†^, diagnostic	y = 0.011*AGE^2 ^- 0.30*AGE - 2.51	0.994
Total intervention rate	y = 0.013*AGE^2 ^- 0.67*AGE + 39.98	0.996
Positive biopsy fraction	y = 0.01*AGE^2 ^+ 0.29*AGE - 21.85	0.992

* Values from Table 1.

^† ^PPV = Positive predictive value.

^‡ ^NPV = Negative predictive value.

Figure 4 shows how the PPVS and PPVD increase with age. The reciprocal of the predicted value, or the number of screening recall mammograms or positive diagnostic mammograms needed to detect a cancer, consequently decreases with age. The average PPVS or CDR/recall rate at age 50 is 4.9% (range 3.3–8.1), and the reciprocal is 20 screening positives/cancer. A radiologist skilled in finding breast cancer but with average specificity would improve PPVS at age 50 to 5.3%, but a radiologist skilled in calling normal as normal but with average sensitivity would have a PPVS of 9.6%. This doubling highlights the primary importance of specificity in improving secondary performance measures.

Figure 5 also shows that the predicted total baseline mammogram intervention rate for women over 50 varies from 39/1000 at age 50 to 51/1000 at age 65. The PBF at age 45 of 10% (range 7–16) is lower than the NBCCEDP (12.8%) and BCSC modified (15.6%) values, while the PBF of 26% at age 55 (range 19–38) is closer to the corresponding values of 19% and 27%. The negative predictive value of a screening mammogram varies from 99.95% at age 40 to 99.93% at age 50 and 99.79% at age 60. The low prevalence and high specificity overwhelm the effect of sensitivity: at age 50 and 10% sensitivity, the negative predictive value drops to only 99.2%.

Table 4 summarizes the actual BCSC cancer rates and selected performance measures as percentages of the model input incidence, prevalence and corresponding model output predictions. The actual BCSC cancer rate at 9–15 months averages 115% of the model input SEER incidence data. However, the actual BCSC first mammography cancer rate varies between 55% and 77% of the model input prevalence, mean 66%. Even when using the low range of sojourn time, which decreases the model input, the mean is 81%. Consequently, the actual BCSC CDR is below the predicted CDR on average about 72% using one-year sensitivity, and 98% using two-year sensitivity. The actual BCSC recall rate averages 96% of the predicted recall rate, which implies the specificity in the model is reasonably accurate. The actual BCSC PPVS averages 71% of the predicted PPVS, again reflecting the lower actual cancer rate.

**Table 4:** Screening mammography BCSC performance data.*

**AGE**	**42.5**	**45**	**47.5**	**50**	**52.5**	**55**	**57.5**	**60**	**62.5**
BCSC CANCER RATE/1000, 9–15 Months^†^	2.25		2.98		3.66		4.44		5.08
Percent of model input incidence^‡^	144		117		109		104		103
BCSC CANCER RATE/1000, No Previous^§^	2.49		4.39		5.73		9.99		10.64
Percent of model input prevalence^‡ ^(high-low)	77 (67–86)		72 (63–83)		59 (51–70)		69 (58–83)		55
BCSC CANCER DETECTION RATE/1000	2.00	3.40^||^	3.75		5.11	8.36^||^	9.32		9.55
% 1 year sensitivity	75	94	75		57	76	70		55
% 2 year sensitivity	102	128	102		78	103	94		75
BCSC RECALL RATE/1000^¶^	123	143^||^	149		151	156^||^	147		132
Percent of model	86	99	103		102	104	96		85
BCSC PPVS**, %	1.6	2.0^||^	2.5		3.3	4.9^||^	6.3		7.2
Percent of model	86	80	73		55	67	72		65

* Data available at Breast Cancer Surveillance Consortium (BCSC) website using screening examinations from 1996–2005.[32]

^† ^Months since previous mammography.[26]

^‡ ^Prevalence and incidence rates are taken from Table 1.

^§ ^No previous mammography or first mammography.[26]

^|| ^Published performance data for 40–49 and 50–59 age groups.[25]

^¶ ^Recall for diagnostic imaging occurs within 90 days after the initial screening exam.

** Positive predictive value of a screening mammogram, which arithmetically equals the cancer detection rate/recall rate.[26]

## Discussion

We have constructed a decision model of screening mammography using population-based parameters that allows reasonable prediction of baseline primary events and secondary performance measures. We found that the recall percentage is generally stable with age at about 15% (range 7–24%), with women facing an overall recall of about 18% (including short-interval follow-up imaging), with a 14% chance of an immediate false-positive exam. Our decision model predicts how the CDR and PBF generally track the increasing prevalence of breast cancer with age: a 50-year-old woman has 3.4 times the prevalence of a 40 year old, while the predicted CDR and PBF are 3.8 and 3.3 times as high, respectively.

Our model predicts how starting screening at age 45 or 55 would considerably improve overall baseline mammography secondary performance measures simply because breast cancer is relatively more common and easier to detect as women age, while recall rates are stable. The increasing CDR means the absolute benefit from baseline screening increases with age, given the potential life-extending benefit from earlier detection and treatment through delaying or preventing advanced disease. However, the recall rate which is mostly unwanted (collateral) false-positive exams in healthy women (95% at age 50, or 1 – PPVS) is relatively stable with age. So the potential benefit/collateral cost ratio gradually increases with age [6], as shown by the increasing PPVS, PPVD and PBF values.

In reality, the major screening problem of overdiagnosis, or the detection of nonprogressive or nonlethal pseudodisease, complicates this simplified economic analysis [21]. Overdiagnosis leads to harmful overtreatment and associated anxiety without any potential benefit to the woman [45,46]. The source of the absolute benefit from screening is the earlier detection of and effective intervention for some otherwise lethal tumors. The absolute risk reduction is equal to the relative risk reduction times the absolute death risk. If the relative risk reduction from screening is constant, the increasing age-dependent absolute death risk determines the absolute benefit [47]. The breast-cancer deaths prevented by screening translated into the age-dependent discounted years of life saved will ultimately reflect the absolute benefit [48]. Consequently, the CDR as a performance measure directly reflects the development risk for breast cancer, but only indirectly reflects the absolute benefit of screening mammography.

Adjusting the CDR to reflect the proportion of lives saved among the women with mammography-detected cancers may present a more realistic benefit. We have estimated this proportion to be under 5% [49,50]. Breast cancer is a heterogeneous disease, with grades of metastatic potential and length of sojourn time [51]. Screening also has a limited window for possible effectiveness and may work only when the detection occurs before the critical point (development of metastases), and if both events occur during the sojourn time [21]. Earlier detection will not extend lives if the critical time point occurs after the onset of breast symptoms, or before the breast cancer is detectable by imaging. In less lethal cancer (including pseudodisease), the critical time point may be delayed or never occur. Detecting a cancer early can also cause harm: earlier intervention associated with mammography appears to activate dormant metastases in some cases, especially in younger women [52].

### Clinical implications

Stratifying the recommendations for screening so that only higher-risk women would be encouraged to screen earlier or more often would increase the prevalence and therefore improve performance measures [53,54]. Recent research on risk stratification for breast cancer shows this approach may be practical to increase screening efficiency [55]. Notably, performance measures are worse in women under age 50: 39% of all diagnostic mammograms after screening occur in this group [40], but less than 25% of invasive cancers do [53]. A screening "cost-effectiveness" proxy is the negative biopsy fraction (1-PBF), since a negative intervention incurs high financial and emotional costs due to its invasive nature without a corresponding potential benefit of cancer detection. Our model predicts that the negative biopsy fraction is over 90% (range 84–93%) for women under 45 and does not approach performance benchmarks of 60–75% [27,40] until after age 55. As shown in Figure 5, even the best United States radiologists will not achieve a level of 75% until women reach age 49.

Consequently, United States organizations [2] and other countries [56,57] have varied opinions regarding the appropriate age to begin and way to practice screening. For instance, the United Kingdom screens women ages 50 to 70 with a screening interval of 3 years, with about half the recall rate but similar cancer detection rates compared with the United States for baseline and subsequent exams [42,58]. Therefore, United Kingdom performance measures should be higher. The European desirable recall rate is under 5% for a baseline exam (and under 3% for a subsequent exam), which is also half the 10% United States guideline for all mammograms [31]. Analysis of BCSC data shows the United States desirable recall rate should be no more than 10% for baseline and 6.7% for subsequent exams [25]. These recommendations and practices may reflect differences in practice environments and radiologist skill. However, using a broader economic perspective, these targets should reflect an analysis of the opportunity cost of resources devoted to screening and diagnosis versus the absolute net benefit of lives saved minus screening harms. Unfortunately, screening advocates have exaggerated this net benefit through the emphasis on relative rather than absolute risk reduction with screening and the failure to discuss mammography harms in many scientific articles [11].

The principle of IMDM decentralizes this debate and helps each woman instead of a "policymaker" or potentially biased "expert" decide what starting age is best for her regarding screening [59]. However, the woman must understand the benefits and harms as well as the true opportunity cost of screening mammography [5,60,61]. Although age is the most important risk factor for breast cancer development, most women are unaware of this fact [62]. Women tend to identify genetics as paramount, yet only 10% of all breast cancer patients have a family history of first-degree relative (mother, sister, and daughter) with cancer [53]. To stress the importance of age, perhaps decision aids could present risk as an equivalent risk-adjusted age for breast cancer development [63]. In this way, a higher risk woman could anticipate mammography performance as equivalent to that for an older woman.

Since the appropriate contents of a screening decision guide are debatable, research is needed on applying these results and making decision guides easier for physicians to present and for women and the public to understand [53,64,65]. For example, a decision guide for younger women utilizes a simplified decision tree of breast-cancer screening [66,67]. Including discussion of DCIS overdiagnosis and the accuracy limitations of mammography, which result in false-positive follow-up testing and associated psychological costs, would improve this type of decision guide [68]. Explaining that "peace of mind" after a negative mammogram [15] is more a function of low prevalence than the sensitivity of the baseline exam would also promote realistic expectations.

### Model applications

Besides being an education tool, we can use our model to predict the effect of new technology on performance measures [69]. For example, the true value of computer-aided detection (CADe) for mammography depends on the overall marginal effect on both CDR, or (sensitivity times prevalence); and the recall rate = (CDR + ([1-specificity] times [1-prevalence])) [49,70]. Our model shows how both the CDR and FP cases/1000 screens influence the other performance measures. For example, the BCSC CADe study showed relative increases in sensitivity (84.0/80.4) and decreases in specificity (87.2/90.2). Using these same relative changes, our model predicts for 50-year-old women an increase of 29% in the recall rate (32% actual), a drop of 19% in the PPVS (22% actual), an increase of 26% in the TIR (20% actual), and a drop of 17% in the PBF [71]. The secondary performance measures will be stable only if there are equal relative percentage changes in both the CDR and FP cases/1000 screens.

Receiver operating characteristic analysis computes the inverse relationship and inherent trade-off between sensitivity (TPF) and specificity (1-FPF) given the natural overlap between diseased and healthy individuals, independent of prevalence. In theory, when prevalence is incorporated and costs of decision consequences are computed, the optimal threshold or sensitivity/specificity pair on any receiver operating characteristic curve can be calculated [30]. We used our model to predict how the recommended threshold for screening mammography varies with age or risk. The optimal operating points appear closer to European than United States recommendations, but depend on multiple assumptions including false-positive exam resource costs and the value assigned to a year of life saved [72].

### Limitations

Our analysis is limited to the decision to undergo a baseline exam, the first step in screening mammography. There is an obvious benefit of higher specificity and reduced false-positive recalls in subsequent mammography when prior mammograms are available, although specificity will also drop as sensitivity increases with decreasing frequency of screening [26]. Complete evaluation of age-related screening performance would require modeling the effects of both initial and repeat screening over multiple periods and frequencies of screening [73]. However, these models must choose an arbitrary period for repeated screening as well as mortality benefit assumptions [74].

Furthermore, a comprehensive model may be less useful for education purposes. Our single mammogram model focusing on performance measures helps quantify the primary importance of age. A woman makes the decision to screen independently each time; a woman cannot buy a coupon worth seven screens over the next decade. The fact is that many women undergo sporadic screening and ignore recommendations. For instance, mammography registry data and models show that although 75% of women age 40 to 50 have obtained a baseline mammogram, over 43% have a gap time of over 2.5 years, and only 24% get an annual mammogram [75,76].

The cancer detection rates for the baseline exam predicted from our model are higher than the actual BCSC cancer detection rates because the model input prevalence is higher: both sensitivity values use the same BCSC data. However, these BCSC and NBCCEDP first mammograms may not be truly baseline and may underestimate true prevalence as shown by the differences in cancer rates. The fact that the 9–15 months actual BCSC data are close to the SEER incidence rates implies that the BCSC has biased data for first mammography, especially for younger women, or that the sojourn estimates are biased. Our sojourn time estimates could be too long; this could occur with overdiagnosis [22]. Furthermore, SEER incidence data is an average and based on all cancer diagnoses, and screened and non-screened women with cancer could have different incident rates. The higher predicted CDR from our model is conservative in that it will result in higher predicted PPV performance measures in Figures 4 and 5.

Consequently, we could improve our model with better estimates of risk-adjustable prevalence and sojourn time, as well as baseline total intervention rates. Although our model also uses older accuracy data that does not include recent digital technology, the effect should be minimal. In one large trial, digital mammography had no overall accuracy benefit compared to film, but digital did have increased sensitivity and receiver operating characteristic curve accuracy benefits in the single subset of women under 50 with dense breasts (17% of participants, digital 1.25 year sensitivity 59% versus film 27%) [77]. But since most digital units have CADe technology [78], any combined digital/CADe claim for accuracy gain is problematic because CADe causes decreased receiver operating characteristic curve accuracy [71]. The excess false-positive recalls due to CADe's influence on the radiologist calling a normal mammogram abnormal (computer-aided deception) overwhelms the benefit (extra cancer detection). As our model predicts, false-positive recall exams should increase substantially with combined digital/CADe mammography [79].

Finally, the CDR is a limited performance measure for radiologists due to the influence of age-related prevalence: as Figure 2 shows, the 90^th ^percentile (highly skilled) radiologist reading mammograms for women age 50 will find fewer cancers than the 10^th ^percentile radiologist reading mammograms for women age 55. Emphasizing the CDR also reinforces the naïve but widely held notion that breast cancer is a homogeneous disease [15], and therefore the belief that earlier detection is always helpful. For instance, in one cross-sectional survey, 94% of women thought that a woman with screen-detected cancer "may have benefited from the mammograms" [14]. Consequently, to support IMDM and professional honesty [80,81], radiologists should continually emphasize to the public that the correct scenario is "earlier cancer detection saves some lives-but screening harms healthy women" or "mammography might extend your life-but often misses cancers."

## Conclusion

By applying decision analysis, we have modeled the consequences of the single baseline screening mammography decision as a function of age and predicted the CDR, recall rate, and various positive predictive values. The principle of IMDM requires that women be educated about mammography accuracy limitations and the substantial age dependence of cancer incidence rates and prevalence, and consequently baseline screening mammography primary and secondary performance measures.

## Abbreviations

*BCSC*: Breast Cancer Surveillance Consortium; *BI-RADS*: Breast Imaging Reporting and Data System; *CADe*: Computer-aided detection; *CDR*: Cancer detection rate, or TP cases/1000 women screened; *D+*: Women with cancer; *D-*: Healthy women; *DCIS*: Ductal carcinoma in situ; *FN*: False-negative; *FNA*: Fine-needle aspiration; *FP*: False-positive; *FPF*: False-positive fraction, or 1 – specificity; *IMDM*: Informed medical decision-making; *NBCCEDP*: National Breast and Cervical Cancer Early Detection Program; *PBF*: Positive biopsy (intervention) fraction;* PPVD*: Positive predictive value diagnostic mammogram after screening; *PPVS*: Positive predictive value screening mammogram; *SEER*: Surveillance Epidemiology and End Results; *TIR*: Total intervention rate, including FNA; *TN*: True-negative; *TP*: True-positive; *TPF*: True-positive fraction, or sensitivity.

## Competing interests

The authors declare that they have no competing interests.

## Authors' contributions

JDK conceived the study, constructed the model, and developed the model inputs. Both JDK and JEK analyzed the model output, and revised and approved the final manuscript.

## Pre-publication history

The pre-publication history for this paper can be accessed here:


